# Dysregulated mTOR networks in experimental sporadic Alzheimer’s disease

**DOI:** 10.3389/fncel.2024.1432359

**Published:** 2024-09-25

**Authors:** Suzanne M. de la Monte, Ming Tong

**Affiliations:** ^1^Departments of Medicine, Pathology and Laboratory Medicine, Neurology, and Neurosurgery, Rhode Island Hospital, Women and Infants Hospital, The Alpert Medical School at Brown University, Providence, RI, United States; ^2^Department of Medicine, Rhode Island Hospital, The Alpert Medical School at Brown University, Providence, RI, United States

**Keywords:** Alzheimer’s disease, mTOR, streptozotocin, rat model, neurobehavior

## Abstract

**Background:**

Beyond the signature amyloid-beta plaques and neurofibrillary tangles, Alzheimer’s disease (AD) has been shown to exhibit dysregulated metabolic signaling through insulin and insulin-like growth factor (IGF) networks that crosstalk with the mechanistic target of rapamycin (mTOR). Its broad impact on brain structure and function suggests that mTOR is likely an important therapeutic target for AD.

**Objective:**

This study characterizes temporal lobe (TL) mTOR signaling abnormalities in a rat model of sporadic AD neurodegeneration.

**Methods:**

Long Evans rats were given intracerebroventricular injections of streptozotocin (ic-STZ) or saline (control), and 4 weeks later, they were administered neurobehavioral tests followed by terminal harvesting of the TLs for histopathological study and measurement of AD biomarkers, neuroinflammatory/oxidative stress markers, and total and phosphorylated insulin/IGF-1-Akt-mTOR pathway signaling molecules.

**Results:**

Rats treated with ic-STZ exhibited significantly impaired performance on Rotarod (RR) and Morris Water Maze (MWM) tests, brain atrophy, TL and hippocampal neuronal and white matter degeneration, and elevated TL pTau, AβPP, Aβ, AChE, 4-HNE, and GAPDH and reduced ubiquitin, IL-2, IL-6, and IFN-γ immunoreactivities. In addition, ic-STZ reduced TL ^pY1135/1136^-IGF-1R, Akt, PTEN, ^pS380^-PTEN, ^pS2448^-mTOR, p70S6K, ^pT412^-p70S6K, p/T-^pT412^-p70S6K, p/T-Rictor, and p/T-Raptor.

**Conclusion:**

Experimental ic-STZ-induced sporadic AD-type neurodegeneration with neurobehavioral dysfunctions associated with inhibition of mTOR signaling networks linked to energy metabolism, plasticity, and white matter integrity.

## 1 Introduction

Alzheimer’s disease (AD) is regarded as one of the most dominant forms of central nervous system neurodegeneration that evolves from clinically normal aging to mild cognitive impairment, followed by progressive neurocognitive dysfunction that culminates with dementia (Tahami Monfared et al., 2022). The neuroanatomical substrate of AD includes a gradual buildup of aggregated and fibrillar brain deposits of Amyloid β (Aβ) and hyperphosphorylated Tau (pau) (de la Monte, 2023). These abnormalities, which are widely considered neurotoxic and pivotal pathogenic factors in AD progression, have been exploited by non-invasive neuroimaging (Roman and Pascual, 2012; Ewers et al., 2011) and cerebrospinal fluid (CSF) diagnostic assays (Wallin et al., 1999; Olsson et al., 2016; Wurtman, 2015; Schaffer et al., 2015), and they have been aggressively targeted for disease remediation (de la Monte, 2023). However, the fundamental hypothesis that Aβ and pTau lesions cause rather than result from AD is not well supported by data showing closely related or identical pathologies in brains with “normal aging,” various forms of neurodegeneration, or incident pathologies such as head trauma, ischemia, metabolic, and neuroinflammatory diseases (de la Monte, 2023; Cunnane et al., 2020; de la Monte, 2014). Furthermore, lesion-targeted therapeutics to retard or remediate the clinical course or neuropathological features of neurodegeneration have repeatedly failed (Plascencia-Villa and Perry, 2023).

Growing evidence highlighting mechanistic roles of dysregulated brain metabolism as critical to AD pathogenesis has broadened our perspective and opened opportunities to address mediators of neurodegeneration that extend beyond Aβ and pTau (de la Monte, 2023). Despite evidence that AD includes a wide range of pathological processes that impact all cell types and extend into most brain regions, there is a dearth of knowledge corresponding to the entire spectrum of disease, particularly the non-Aβ and non-pTau abnormalities such as white matter atrophy and degeneration, glial, microvascular, and microglial pathologies, and dysregulation of energy metabolism (de la Monte, 2023; Bloom, 2014). Of note is that all these non-Aβ and non-pTau abnormalities were recognized for years but cast aside due to intensive research on the cloning and molecular characterization of Aβ and pTau.

Interest in the role of brain metabolic dysregulation as a mediator of AD and other neurodegenerative diseases was kindled by studies demonstrating impairments in brain glucose uptake and utilization, signal transduction through the insulin and insulin-like growth factor (IGF) networks, astrocyte, microvascular, and mitochondrial functions, and the regulation of neuroinflammatory mediators (Cunnane et al., 2020). The contributions of insulin/IGF-1 deficiency and resistance were further spotlighted by epidemiologic links between AD and systemic disorders of insulin resistance including Type 2 diabetes mellitus, obesity, and metabolic fatty liver disease (de la Monte, 2014; de la Monte and Wands, 2005; Hoyer, 1998; de la Monte, 2012a; de la Monte, 2012c; de la Monte, 2012b; de la Monte, 2017; de la Monte, 2019; de la Monte et al., 2009a). Understanding how insulin and IGF regulate brain energy metabolism, neuronal plasticity, oligodendrocyte functions including myelin maintenance, anti-oxidant, cell survival, and homeostatic mechanisms, and cytoskeletal functions could facilitate predicting the effects of trophic factor withdrawal and receptor resistances, and enhance appreciation of the molecular pathologic parallels between AD and diabetes mellitus (de la Monte and Grammas, 2019). Finally, brain insulin resistance has been linked to astrocyte and microglia dysfunctions that promote tau hyperphosphorylation and reduce Aβ degradation, causing neurotoxic paired-helical filament (PHF) and Aβ lesions to accumulate, exacerbate neuroinflammation, and drive the cascade of neurodegeneration (de la Monte, 2023; Chen et al., 2023).

Studies on the roles of dysregulated and compromised insulin and IGF-1 networks in AD, both humans and experimental models, revealed that neurodegeneration is associated with disease severity-related reductions in the expression of both the trophic factors and receptors (Rivera et al., 2005; Robbins et al., 2020; Steen et al., 2005; Talbot et al., 2012). Due to the combined features reminiscent of both Type 1 diabetes mellitus and Type 2 diabetes mellitus, the term “Type 3 Diabetes” was coined to provide a conceptual framework regarding the dominant mechanisms of neurodegeneration (de la Monte, 2014; Steen et al., 2005). Investigations in many laboratories showed that with the functional reductions in trophic factors and corresponding receptors, signals transmitted downstream through the insulin receptor substrate (IRS) docking proteins (de la Monte, 2014; Hoyer, 1998; de la Monte, 2012a; de la Monte, 2012b; de la Monte, 2017; de la Monte, 2019; de la Monte et al., 2009a; Chen et al., 2023; Steen et al., 2005; Talbot et al., 2012; Akhtar and Sah, 2020; Gabbouj et al., 2019; Hoyer, 2004; Lester-Coll et al., 2006; Salkovic-Petrisic et al., 2006; Sędzikowska and Szablewski, 2021), phosphatidylinositol-3-kinase (PI3K), Akt, and glycogen synthase kinase 3β (GSK-3β) were consistent abnormalities (Gabbouj et al., 2019). The inhibition of Akt has relevance to AD-associated deficits in brain energy metabolism, growth, cell survival, and neuronal plasticity. GSK-3β activation associated with inhibition of PI3K-Akt is a key driver of pro-oxidant and pro-stress pathways, neuroinflammation, Tau phosphorylation, and anti-cell survival, together leading to increased Aβ aggregation and PHF pathology (Salkovic-Petrisic et al., 2006; Beurel et al., 2015; Han et al., 2016; Phiel et al., 2003; Toral-Rios et al., 2020). Signaling through the Akt serine/threonine kinase is of particular interest because of its stimulatory support of growth, metabolism, and cell survival in neurons and oligodendrocytes (Hers et al., 2011), and the fact that inhibition of Akt impairs oligodendrocyte functions, including myelination (Figlia et al., 2018; Narayanan et al., 2009). Ultimately, the functional integrity of neurons and oligodendrocytes is integrally linked to the mechanistic target of rapamycin (mTOR) networks that are responsive to cellular nutrition, growth factor stimulation, e.g., insulin/IGF-1, and energy levels (Rosner et al., 2008; Tian et al., 2019) and regulates many functions including memory, cognition, and synaptic plasticity, glial functions, cellular homeostasis and myelination (Bockaert and Marin, 2015; Ishizuka et al., 2008; Lee, 2015), all of which are relevant to AD (de la Monte, 2023).

mTOR signaling is effectuated via its molecular complexes, mTORC1 and mTORC2, in which mTOR interacts with Raptor, TSC, mLST8, PRAS40, and DEPTOR (mTORC1) (Tian et al., 2019; Rosner et al., 2010), or Rictor, Protor-1/2, mammalian stress-activated MAP kinase-interacting protein 1 (mSIN1), mLST8 and DEPTOR (mTORC2) (Tian et al., 2019; Bockaert and Marin, 2015). The selectivity and specificity of mTORC1 and mTORC2 are conferred in part by differences in their subcellular localizations that enable substrate activation or inhibition (Graber et al., 2013). Serine phosphorylation of mTOR marks its activated state (Graber et al., 2013; Copp et al., 2009), but its specificity for downstream targets is dictated by site-dependent phosphorylation. Ser-2448 phosphorylated mTOR binds to Raptor, resulting in mTORC1 kinase activation of p70S6K (Rosner et al., 2010), whereas Ser-2481 phosphorylation (Copp et al., 2009) promotes mTOR’s interaction with Rictor and activation of mTORC2 kinase (Copp et al., 2009; Chiang and Abraham, 2005). p70S6K phosphorylates and activates ribosomal protein S6 (RPS6) (Arif et al., 2019). Activated RPS6 has functional roles in regulating growth, neuronal activity, and synaptic plasticity (Biever et al., 2015). Therefore, the active (phosphorylated) forms of p70S6K and RPS6 reflect mTORC1 activation (Copp et al., 2009; Chiang and Abraham, 2005).

Insulin/IGF-1 activation of mTOR/mTORC1/2 has broad cellular effects that promote functions including growth, nutrient metabolism, proliferation, RNA translation (Lee, 2015), lipogenesis, and lipid storage (Yoon, 2017). The functional outcomes of mTORC1/2 stimulation promote mitochondrial function, re-organization of the actin cytoskeleton, cell motility, cell migration, and metabolism of lipids and proteins and reduce autophagy (Rosner et al., 2010; Bartolome et al., 2014). Within the nervous system, the regulatory effects of mTOR/mTORC1/2 mediate critical neuronal functions needed for cognition and behavior, including development, plasticity, and memory (Graber et al., 2013). Akt’s pivotal role in phosphorylating and activating mTORC2 (Rosner et al., 2008; Rosner et al., 2010) is needed to modulate cell growth, neuronal synaptic plasticity (Ishizuka et al., 2008), glial cell development, white matter and peripheral nerve myelination, and complex processes involved in cognition, behavior, and the establishment of memory (Bockaert and Marin, 2015; Lee, 2015).

In AD, there is now strong evidence that metabolic dysregulation linked to impairments in CNS signaling through insulin/IGF-1 pathways needed to drive PI3K-Akt, mimic the molecular and biochemical pathologies described for diabetes mellitus and similar insulin-resistance disease states (Akhtar and Sah, 2020). Impairments in mTOR signaling, as downstream mediators of neurodegeneration (Akhtar and Sah, 2020; Hoeffer and Klann, 2010; de la Monte et al., 2017), have been implicated based on the corresponding deficits in energy metabolism, mitochondrial function, cell growth and survival, synaptic plasticity, and dysregulated autophagy. Correspondingly, in human brains with AD and experimental models with AD-type neurodegeneration, mTORC1 and mTORC2, their related enzymatic activities (Lee et al., 2017), and their downstream targets (de la Monte et al., 2017) are all inhibited. Eukaryotic factors of initiation (EIFs) utilize ds RNA-dependent protein kinase (PKR), which shuts down protein synthesis and is up-regulated in AD human and experimental models. Under those conditions in AD cellular and animal models, mTOR’s regulatory effects on translation are down-regulated (Morel et al., 2009). In contrast, short-term *in vitro* models of AD exhibit mTORC1 inhibition of autophagy and *de novo* protein synthesis (Lee et al., 2017) suggesting the outcomes of mTORC signaling are modulated by disease duration and pathogenic factors.

This study characterizes insulin/IGF-1 receptor-Akt-mTOR-mTORC1/2 signaling networks in an established experimental model of sporadic AD. The model was generated by intracerebroventricular injection of streptozotocin (ic-STZ) which produces cognitive and motor deficits with brain atrophy and increased expression of AD biomarkers (Lester-Coll et al., 2006; Salkovic-Petrisic et al., 2006; de la Monte et al., 2017; Moreira-Silva et al., 2019; Tong et al., 2016a). We anticipated that the new information generated would increase understanding of how long-term impairments in insulin and/or IGF-activated pathways contribute to the diverse neuropathological processes that occur in AD and possibly uncover endogenous adaptive processes that support remaining functions despite chronicity of neurodegeneration.

## 2 Materials and methods

### 2.1 Materials

Streptozotocin (STZ) was purchased from MilliporeSigma (Burlington, MA, USA). Table 1 lists the epitope-specific commercial primary antibodies used in duplex enzyme-linked immunosorbent assays (ELISAs), along with the antibody Research Resource Identifier (RRID) numbers, vendor sources, and experimental conditions. ThermoFisher Scientific (Bedford, MA, USA) was the commercial source for the Bicinchoninic acid (BCA) reagents, ELISA plates (MaxiSorp 96-well), secondary horseradish peroxidase (HRP)-conjugated antibodies, and blocking solution (Superblock TBS) for masking non-specific binding sites. The soluble fluorophores, Amplex Ultra Red and 4-methylumbelliferyl phosphate (4-MUP), were purchased from Life Technologies (Carlsbad, CA, USA). Vector Laboratories Inc. (Newark, CA, USA) was the source for Protein Biotin Protein Labeling Kit and Alkaline Phosphatase-conjugated Streptavidin. The 5-Plex MILLIPLEX MAP Rat Cytokine and the 11-Plex MILLIPLEX MAP Akt/mTOR Total and Phosphoprotein Magnetic Bead Panels were purchased from Millipore (Burlington, MA, USA). All other experimental reagents were purchased from Sigma-Aldrich Co. (St. Louis, MO, USA), Calbiochem/Millipore Sigma (Burlington, MA, USA), and Pierce Chemical (Dallas, TX, USA).

**TABLE 1:** Commercial antibodies and sources.

Antibody targets	Source	Monoclonal/ polyclonal	Stock (mg/ml)	μ g/ml or dilution	Commercial source	RRID# or reference*
AβPP (amyloid β-precursor protein)	Rabbit	Polyclonal	0.197	0.0985	Cell Signaling, Danvers, MA, USA	Cat. #2452
Aβ (amyloid β peptide, 1–42)	Mouse	Monoclonal	0.2	0.8	Santa Cruz Biotechnology, Dallas, TX, USA	Cat. #sc-28365
Tau (tubulin-associated unit)	Rabbit	Polyclonal	6.2	3.1	Agilent/Dako, Santa Clara, CA, USA	REF A0024; Lot# 00033544
pTau (PHF; S396) (phosphorylated tubulin associated unit)	Mouse	Monoclonal	1.21	0.40	Cell Signaling, Danvers, MA, USA	#9632
ChAT (choline acetyltransferase)	Rabbit	Polyclonal	100 uL	1:3000	Abcam, Boston, MA, USA	AB_2244866
AChE (acetylcholinesterase)	Mouse	Monoclonal	1.0	0.25	Abcam, Boston, MA, USA	AB_303316
HNE (4-hydroxy-2-nonenal)	Goat	Polyclonal	0.8	1.6	Abcam, Boston, MA, USA	ab46544
8-OHdG (8-hydroxydeoxy-guanosine)	Mouse	Monoclonal	0.1	0.2	Abcam, Boston, MA, USA	ab48508
Ubiquitin	Rabbit	Polyclonal	0.25	0.5	Abcam, Boston, MA, USA	ab7780-500
GAPDH (glyceraldehyde-3-phosphate dehydrogenase)	Mouse	Monoclonal	200	0.2	Santa Cruz, Dallas, TX, USA	AB_10847862
Rictor	Rabbit	Polyclonal		1:3000	ThermoFisher (Invitrogen, Waltham, MA, USA)	PA5-102842
pSer1591-Rictor	Rabbit	Polyclonal		1:800	ThermoFisher (Invitrogen, Waltham, MA, USA)	PA105669
Raptor	Mouse	Monoclonal		1:1000	ThermoFisher-Bioss, (Woburn, MA, USA)	/BSM-51285M
pSer792-Raptor	Rabbit	Polyclonal		1:2000	ThermoFisher (Bioss)	BS-3381R
RPLPO (large ribosomal protein)	Rabbit	Polyclonal	0.1	0.1	Prointech Group, Inc., Chicago, IL, USA	RPL23 16086-1-AP

*RRID, research resource identifier; references refer to literature citations with validations of RPLPO antibody.

### 2.2 Model for *in vivo* experiments

Long Evans postnatal day 3 (P3) rat pups from Charles River Laboratories (Wilmington, MA, USA) were administered STZ (0.8 mg/kg) or saline (control) by intracerebroventricular (ic) injection as previously reported (Hoyer, 1998; Lester-Coll et al., 2006; de la Monte et al., 2017). On P17, the rats were rotarod tested with 10 progressive speed (2 to 10 rpm) trials in which the latencies to fall were recorded with an automated photocell (Rotamex-5, Columbus Instruments, Columbus, OH, USA). However, to prevent fatigue, the trials were terminated after 45 s and the rats were rested for 10 min between trials. The area-under-curve latencies-to-fall calculated based on the 10 rotarod testing speeds, were used for inter-group comparisons. Morris Water Maze (MWM) testing of spatial learning was performed from P24 through P27. Performance was tracked using Ethovision 8.5 (Noldus, Leesburg, VA, USA) as described (Lester-Coll et al., 2006). The latency tied to locating and landing on the hidden platform was the main performance index used for the inter-group comparisons. To avoid exercise fatigue, the maximum trial duration was capped at 120 s and the rats were rested for at least 5 min between trials. The calculated area-under-curve for recorded latencies over the 4 consecutive days of 3 daily trials per rat were used for inter-group comparisons. Previous studies demonstrated that ic-STZ does not significantly impact swimming speed and that performance differences were due to increased errors and path complexities (Tong et al., 2016b) (Supplementary Figure 1).

The rats were monitored daily for weight gain, feeding, behavior, and general health. The experiments were terminated using isoflurane. The freshly harvested temporal lobes were processed for subsequent analyses. For histopathologic studies, a coronal plane 3-mm slice through the cerebral hemispheres, including temporal lobes and infundibulum, was immersion-fixed in formalin and paraffin-embedded. Dewaxed, rehydrated histological sections (5 μm thick) were histochemically stained with Luxol fast blue, hematoxylin, and eosin (LHE) to reveal neuronal and glial cytomorphology and white matter fibers. The remaining unfixed temporal lobe (TL) tissue was rapidly frozen on dry ice and stored at −80°C. The analytical studies targeted the TL because the temporal cortex is a major target of AD neurodegeneration accompanied by expression of the signature biomarkers, neuroinflammation, and altered integrity of insulin/IGF signaling through the Akt-mTOR pathways (Longato et al., 2012).

The Institutional Animal Care and Use Committee (IACUC) at Lifespan Medical Center approved the use of rats in this research (Committee #500324; approved 02/29/2024). All procedures were done in accordance with guidelines published in the National Institutes of Health (NIH) Guide for the Care and Use of Laboratory Animals.

### 2.3 Tissue homogenization for biochemical studies

Fresh frozen TL tissue samples (50 mg each) from 8 control (4 male and 4 female) and 11 ic-STZ (5 male and 6 female) rats per group were homogenized in weak lysis buffer [50 mM Tris (pH 7.5), 150 mM NaCl, 5 mM EDTA (pH8.0), 50 mM NaF, 0.1% Triton X-100] containing protease inhibitor cocktail (1 mM PMSF, 0.1 TPCK, 2 μg/ml pepstatin A, 1 μg/ml leupeptin, 1 mM NaF, 1 mM Na_4_P2O_7_) and 10 mM Na_3_VO_4_ as a phosphatase inhibitor. A TissueLyser II instrument (Qiagen, Germantown, MD, USA) equipped with 5 mm stainless steel beads was used for tissue homogenization. After centrifuging the samples at 14,000 × *g* for 15 min at 4°C, the supernatants were collected for storage at −80°C and later used in immunoassays. Aliquots were used to measure protein concentration with the BCA.

### 2.4 Duplex ELISA

Duplex ELISAs measured immunoreactivity to Tau (tubulin associated unit), pTau (phospho tau-NFT), amyloid-β-precursor protein (AβPP), the Aβ 1-42 cleavage fragment of AβPP (Aβ), choline acetyltransferase (ChAT), acetylcholinesterase (AChE), glucose-6-phosphate dehydrogenase (GAPDH), ubiquitin (UBQ), 4-hydroxynonenal (4-HNE), 8-hydroxy-2′-deoxyguanosine (8-OHdG), rapamycin-insensitive companion of mammalian target of rapamycin (Rictor), phospho-Rictor (^p Ser1591^Rictor), regulatory-associated protein of mTOR (Raptor), and phospho-Raptor (^p Ser792^Raptor). In brief, triplicate 50ng protein aliquots in 50 μl bicarbonate binding buffer were adsorbed to the bottom surfaces of ELISA plates (96-well) at 4°C overnight. Non-specific sites were masked with Superblock (TBS). Primary antibodies (0.2–5.0 μg/mL) were incubated overnight at 4°C with platform rotation. Species-specific horseradish peroxidase (HRP)-conjugated secondary antibodies and Amplex UltraRed soluble fluorophore detected immunoreactivity. Fluorescence intensity was measured in a SpectraMax M5 microplate reader (*Ex530nm/Em590nm*). Large ribosomal protein (RPLPO) served as a loading control (Longato et al., 2012; Tong et al., 2017; de la Monte et al., 2023a). RPLPO was detected in the same samples with biotinylated antibody, streptavidin-conjugated alkaline phosphatase, and 4-MUP fluorophore (*Ex360 nm/Em450 nm*). Relative levels of immunoreactivity derived from the calculated ratios of target protein to RPLPO immunoreactivities were used for intergroup statistical comparisons.

### 2.5 Multiplex ELISAs

The mTOR signaling pathway was evaluated with 11-Plex MILLIPLEX Akt/mTOR Total and Phosphoprotein Magnetic Bead Kits (MilliporeSigma, Burlington, MA, USA) (Table 2). Neuroinflammatory responses were examined using the 5-plex MILLIPLEX MAP Rat Cytokine/Chemokine Magnetic Bead Panel that included antibodies to interleukin-1β (IL-1β), IL-2, IL-6, Interferon-gamma (IFN-γ), and tumor necrosis factor-alpha (TNF-α). Multiplex ELISAs were performed according to the manufacturer’s protocol. However, the reactions were optimized by using 12.5 μg protein/sample in the 11-Plex Akt/mTOR assays and 150 μg protein/sample for the 5-plex Cytokine/Chemokine ELISA. Following incubation with antibody-bound beads, immunoreactivity in the captured antigens was detected with biotinylated secondary antibodies and phycoerythrin-conjugated streptavidin and measured in a MAGPIX with xPONENT software. Standard curves were generated for each analyte.

**TABLE 2:** Commercial multiplex ELISA platforms.

Antibody targets: total proteins	Antibody targets: phospho-proteins
Akt (protein kinase B)	Akt (473)
GSK-3α (glycogen synthase kinase 3α)	GSK-3α (Ser21)
GSK-3β (glycogen synthase kinase 3β)	GSK-3β (Ser9)
IGF-1R (insulin-like growth factor receptor, type 1)	IGF-1R (Tyr1135/1136)
IR (insulin receptor)	IR (Tyr1162/1163)
IRS1 (insulin receptor substrate, type 1)	IRS1 (Ser636)
PTEN (phosphatase and tensin homolog)	PTEN (Ser380)
RPS6 (ribosomal protein S6)	RPS6 (Ser235/236)
TSC2 (tuberous sclerosis complex 2)	TSC2 (Ser939)
mTOR (mechanistic target of rapamycin)	mTOR (Ser2448)
p70S6K (phospho-p70 S6 kinase)	p70S6K (Thr412)

Protein and phospho-protein targets in the 11-Plex MILLIPLEX Akt/mTOR total and phosphoprotein magnetic bead kits (MilliporeSigma, Burlington, MA, USA). The left column lists the standard molecule abbreviations along with their full names in parenthesis. The right column indicates the specific phosphorylation sites (in parenthesis) corresponding to the target molecules assayed in the phospho-protein 11-plex ELISA.

### 2.6 Statistical analyses

All experiments and assays included 8–10 rats per group. Biochemical and molecular assays were performed in triplicate and repeated twice. The samples were coded for blind assay performance and analysis. Statistical analyses of ic-STZ effects were performed, and graphs were generated with GraphPad Prism 10.2 software (Boston, MA, USA). Area-under-curve analysis determined the effects of ic-STZ on Rotarod and Morris Water Maze test performance. Student *t*-tests with corrections for multiple comparisons assessed the effects of ic-STZ on temporal lobe expression of AD biomarkers, stress molecules, cytokines, and Akt-mTOR pathway molecules. Boxplots depict the means (horizontal bars), 95% confidence interval limits (upper and lower box boundaries), and range (upper and lower stems). Software generated statistically significant (*p* ≤ 0.05) and trend-wise (0.05 < *p* < 0.10) differences are displayed within the graph panels. The “trend-wise *p*-values” are noted only as potentially bordering on significance. However, they are nonsignificant (Nead et al., 2018) and therefore must be regarded with caution.

## 3 Results

**FIGURE 1:**
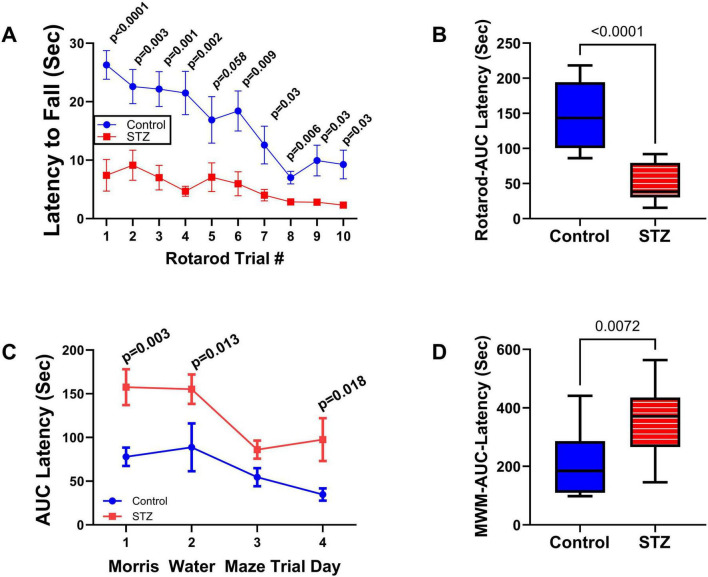
Functional neurobehavioral impairments caused by ic-STZ. Control (*n* = 8) and ic-STZ-treated (*n* = 11) Long Evans rats were subjected to rotarod and Morris water maze (MWM) tests. For rotarod tests, the rats were administered 10 consecutive trials of progressively increasing speed. The latency-to-fall was recorded automatically. **(A)** The graph depicts the mean (± SD) latencies per trial. Results were analyzed by two-way ANOVA with *post-hoc* Tukey tests. **(B)** Area-under-curve (AUC) was calculated over the 10 trials for each rat. The inter-group comparison was made by *t*-test. **(C)** Morris water maze results correspond to the latencies required for the rats to reach and land on the platform. The AUCs were calculated based on the 3 daily trials. Each inter-group Trial Day performance (mean ± SD of the AUC) was compared by *t*-test. **(D)** The AUCs calculated for performance over the 4 trial days per rat were compared to reflect the overall effects of ic-STZ on spatial learning and memory.

Neurobehavioral tests: In previous studies, we conducted neurobehavioral rotarod and Morris water maze (MWM) tests to evaluate motor and spatial learning and memory impairments in the ic-STZ model (Tong et al., 2016a; Tong et al., 2016b). Correspondingly, in the present study, the ic-STZ treatments significantly impaired performance on both the rotarod and MWM tests (Figure 1). Concerning the rotarod test, the ic-STZ-treated rats exhibited significantly shorter mean latencies to fall from the rotating rod in all 10 trials (Figure 1A). Area-under-curve analysis demonstrated significantly worse overall performance due to the nearly 60% reduction in the mean latency-to-fall in the ic-STZ-treated group (Figure 1B). On all 4 MWM trial days, the mean AUC latency to locate and land on the platform was longer for the ic-STZ treated rats, with significant differences from controls on Trial Days 1, 2, and 4 (Figure 1C). In addition, the overall mean AUC latency was significantly greater for the ic-STZ group (*p* = 0.0072) (Figure 1D). There were no significant male versus female within-group differences in rotarod or MWM test performances. The observed ic-STZ impairments in rotarod and MWM performance correspond with previous observations in this model (Tong et al., 2016a).

**FIGURE 2:**
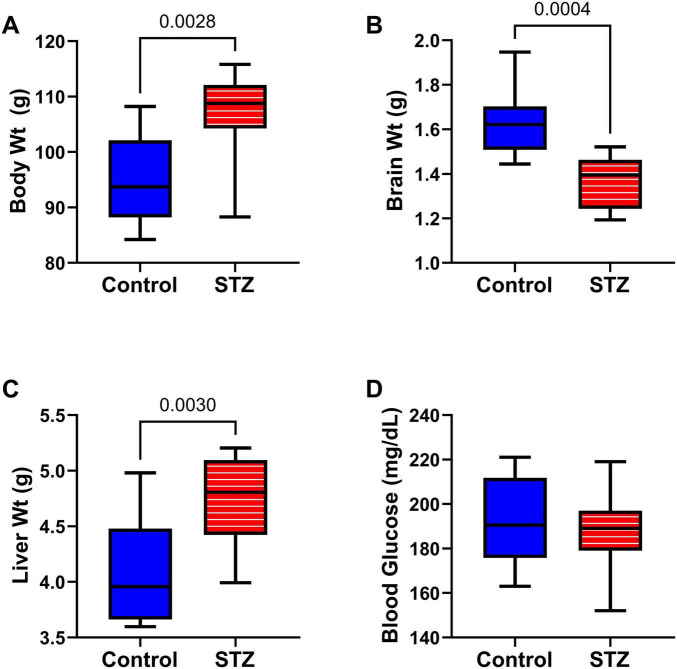
Alterations in body, brain, and liver weight following ic-STZ. Terminal **(A)** body, **(B)** brain, and **(C)** liver weights and **(D)** blood glucose levels were measured upon sacrifice on P30. Inter-group comparisons (*n* = 8 control and 11 ic-STZ samples/group) were made by repeated measures *t*-tests.

Effects of ic-STZ on body, brain, and liver weights, and blood glucose: Through P27, rats in the control and ic-STZ groups had similar body weights and exhibited good health. At the time of rotarod testing, the mean (± SD) of body weight was 55 ± 2.96 for controls and 49.7 ± 8.09 for the ic-STZ group (Not significant by *t*-test analysis; *p* = 0.10). At the time of MWM testing, the mean (± SD) of body weight was 73.3 ± 4.96 for the control group and 68.3 ± 9.2 for the ic-STZ group (Not significant by *t*-test analysis; *p* = 0.18). However, subsequent responses to the ic-STZ treatments were manifested by significant alterations in the body, brain, and liver weights (Figure 2). Corresponding with the consequences of dysregulated metabolism, the mean body (Figure 2A) and liver (Figure 2C) weights were significantly increased, whereas brain weight was reduced (Figure 2B) by ic-STZ. In contrast, blood glucose was not significantly altered by ic-STZ (Figure 2D). These findings correspond with previous observations in this model (Lester-Coll et al., 2006; Tong et al., 2016b).

**FIGURE 3:**
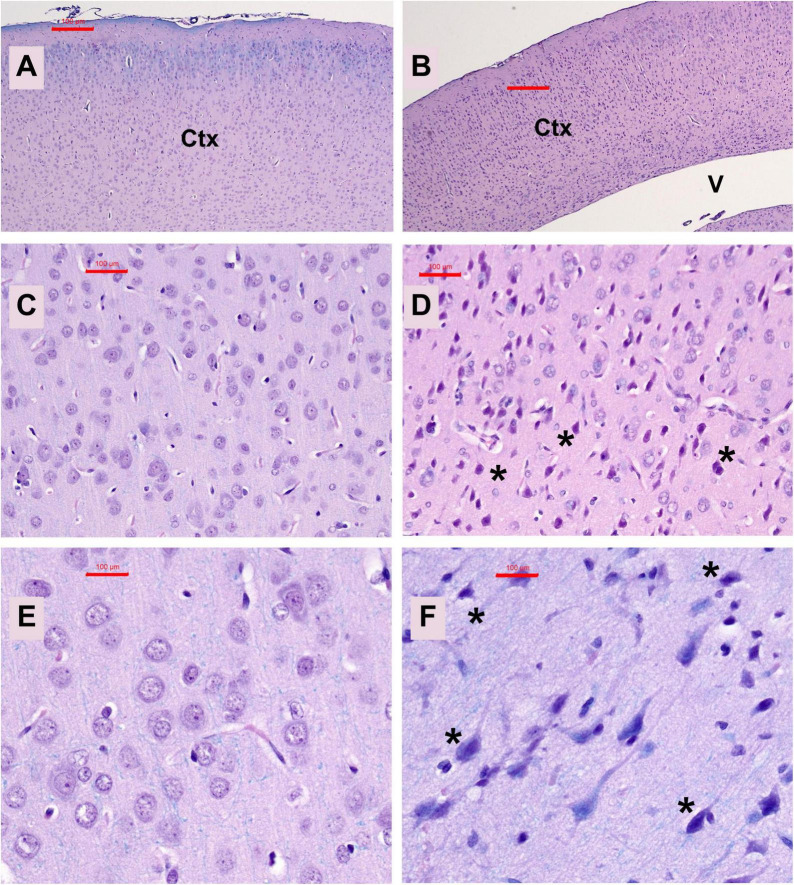
Temporal cortex pathology. Formalin-fixed, paraffin-embedded histological sections (5 μm thick) of the temporal lobe from control **(A,C,E)** and ic-STZ-treated **(B,D,F)** P30 rats were stained with Luxol fast blue, hematoxylin, and eosin (LHE). Note thinning of the cortex (Ctx) accompanied by enlargement of the lateral ventricle (V) in the ic-STZ low-magnification image **(B)** compared with a representative control **(A)** in which the ventricle is not visible due to the thickness of the cortex. Higher magnification images display numerous dark, pyknotic neurons [asterisks in **(D,F)**] compared with uniformly round to oval neuronal nuclei in the control brain **(C,E)**. Original magnifications: **A,B** = 20×; **C,D** = 100×; **E,F** = 400×; Scale bars correspond to 100 μm.

**FIGURE 4:**
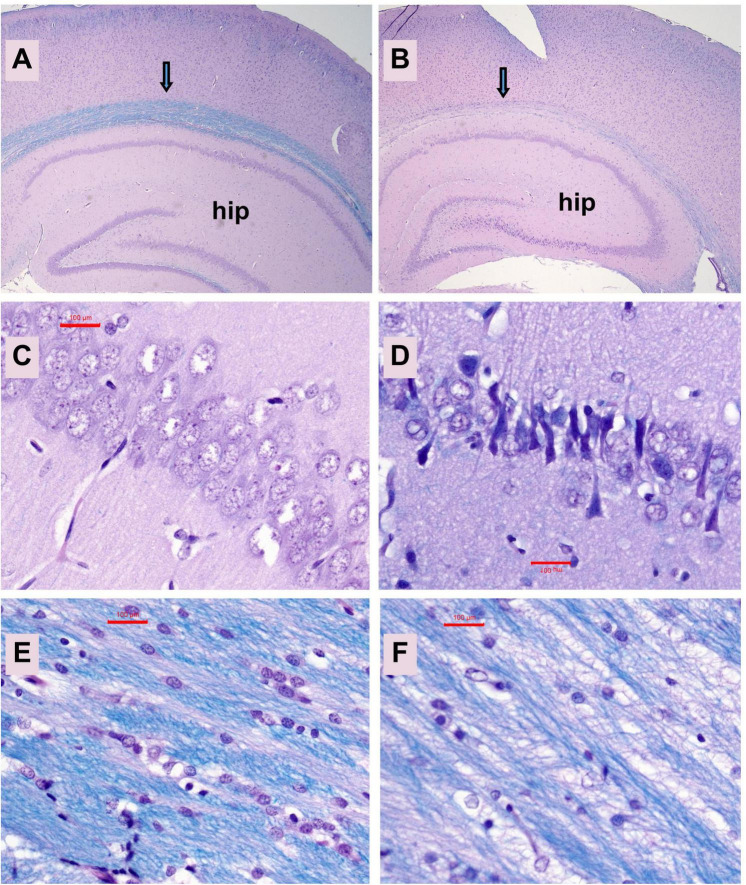
Hippocampal atrophy. Formalin-fixed, paraffin-embedded histological sections (5 μm thick) of the temporal lobe with hippocampal formation from control **(A,C,E)** and ic-STZ-treated **(B,D,F)** P30 rats were stained with LHE. Note the smaller size of the hippocampus (hip) and pallor of the white matter track (arrow) overlying the hippocampus in the ic-STZ case. **(B,D)** CA1 region of the hippocampus demonstrates striking neuronal degeneration (darkening and shrinkage of neurons and narrowing of the zone) in the **(D)** ic-STZ versus **(C)** control. **(E,F)** White matter pallor, cell loss and vacuolation in the ic-STZ compared with control. Note the lower density of nuclei (glial cells-oligodendrocytes) and bubbly white vacuolation of myelin corresponding to myelin and fiber loss due to ic-STZ. Original magnifications: **A,B** = 20×; **C–F** = 400×. Scale bars correspond to 100 μm.

Neuropathological changes in the ic-STZ model: Paraffin-embedded histological sections of temporal lobe including the hippocampal formation demonstrated that ic-STZ caused atrophy (thinning) of the cortical ribbon with contraction of the neuropil rendering an appearance of increased cellular crowding. Cortical atrophy was accompanied by enlargement of the ventricle, and abundant neurons with shrinkage and condensation or apoptosis (Figures 3B, D, F). These appearances contrasted with the uniformly distributed round-to-oval neuronal nuclei with prominent nucleoli in the control temporal cortex (Figures 3A, C, E). Sections through the hippocampal formation, including Ammon’s horn and the overlying cortex, revealed an overall larger hippocampus and white matter with more conspicuous and intense Luxol Fast blue staining in control versus ic-STZ samples (Figures 4A, B). Higher magnification images of the CA1 hippocampal region revealed a uniform linear organization of intact neurons in controls (Figure 4C) and thinning with abundant hyperchromatic (darkened) shrunken neurons in the ic-STZ cases (Figure 4D). Pallor of white matter staining in the ic-STZ temporal lobes was associated with reduced intensity of Luxol fast blue staining, less abundant glial cells (mainly oligodendrocyte), and vacuolation of tissue reflecting fiber loss (Figure 4F) relative to the uniform blue staining with abundant glial nuclei and minimal vacuolation in control samples (Figure 4E).

**FIGURE 5:**
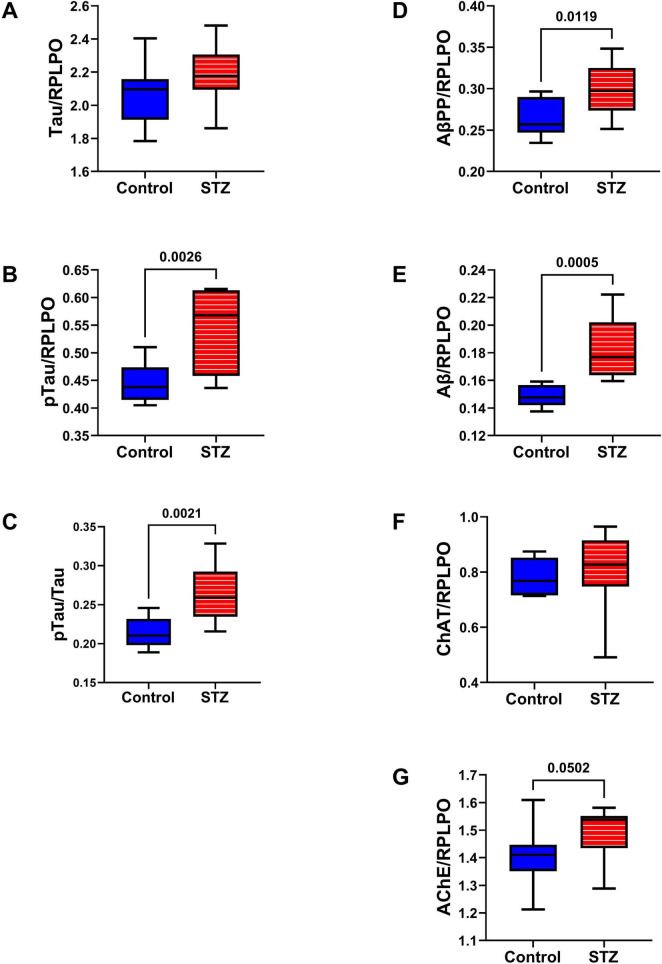
AD biomarker expression. Duplex ELISAs measured temporal lobe immunoreactivity to **(A)** Tau, **(B)** pTau (PHF), **(C)** pTau/Tau, **(D)** AβPP, **(E)** Aβ **(F)** ChAT, and **(G)** AChE with results normalized to RPLPO. The control group included 8 rats and the ic-STZ group included 11 rats. Inter-group statistical analyses were by repeated measures *t*-tests. Significant *p*-values (*p* ≤ 0.05) are displayed within the panels.

AD Biomarkers: The effects of ic-STZ on temporal lobe expression of tau, pTau, AβPP, Aβ, ChAT, and AchE were evaluated by duplex ELISA in which target molecule immunoreactivity was normalized to RPLPO (Figure 5). In addition, the pTau/Tau ratios for each sample were calculated and analyzed statistically. The ic-STZ treatments resulted in significantly elevated levels of pTau (Figure 5B), pTau/Tau (Figure 5C), AβPP (Figure 5D), Aβ (Figure 5E), and AChE (Figure 5G) but not total Tau (Figure 5A) or ChAT (Figure 5F). Therefore, ic-STZ increased the expression of neurofibrillary tangle-related pTau, and both AβPP and its Aβ(1–42) cleavage product, corresponding with the findings in AD. In addition, the elevated levels of AChE relate to the known loss of cholinergic function in AD, as previously observed in this animal model (García-Ayllón et al., 2011).

**FIGURE 6:**
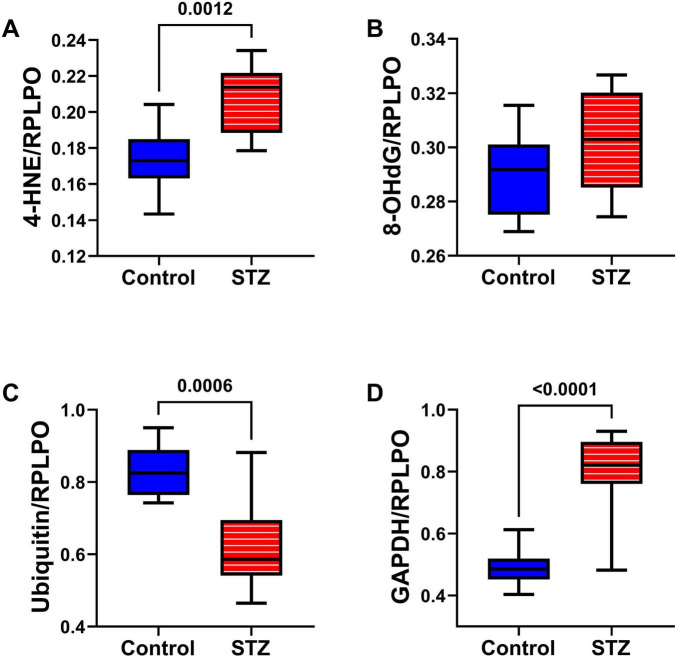
Oxidative stress markers. Temporal lobes from Long Evans rats treated with ic saline (Control, *n* = 8) or STZ (*n* = 11) were analyzed by duplex ELISA to measure **(A)** 4-HNE, **(B)** 8-OHdG, **(C)** Ubiquitin, and **(D)** GAPDH with results normalized by RPLPO. Inter-group comparisons were made by repeated measures *t*-tests. Significant differences (*p* ≤ 0.05) are displayed.

Oxidative Stress Molecules: Duplex ELISAs measured HNE (Figure 6A), 8-OHdG (Figure 6B), ubiquitin (Figure 6C), and GAPDH (Figure 6D) to assess long-term effects of ic-STZ on indices of oxidative stress (de la Monte et al., 2011; Tong et al., 2012). HNE, a marker of lipid peroxidation, was significantly increased in the ic-STZ brains (Figure 6A). The modest ic-STZ-associated elevation of 8-OHdG, a marker of DNA damage, did not reach statistical significance. Ubiquitin, which frequently binds to abnormally aggregated and misfolded proteins in AD and other neurodegenerative diseases (Amzallag and Hornstein, 2022; Behl et al., 2022; Tecalco-Cruz et al., 2022; Tokunaga and Ikeda, 2022), was significantly reduced following ic-STZ. However, extreme oxidative stress can inactivate the proteasome, inhibiting ubiquitination via reductions in newly formed ubiquitin conjugates (Shang and Taylor, 2011). GAPDH was significantly increased in ic-STZ-treated brains. Paradoxically, GAPDH expression in the brain was previously shown to be elevated under conditions of increased oxidative stress and neuronal damage (Hwang et al., 2007; Ou et al., 2010).

**FIGURE 7:**
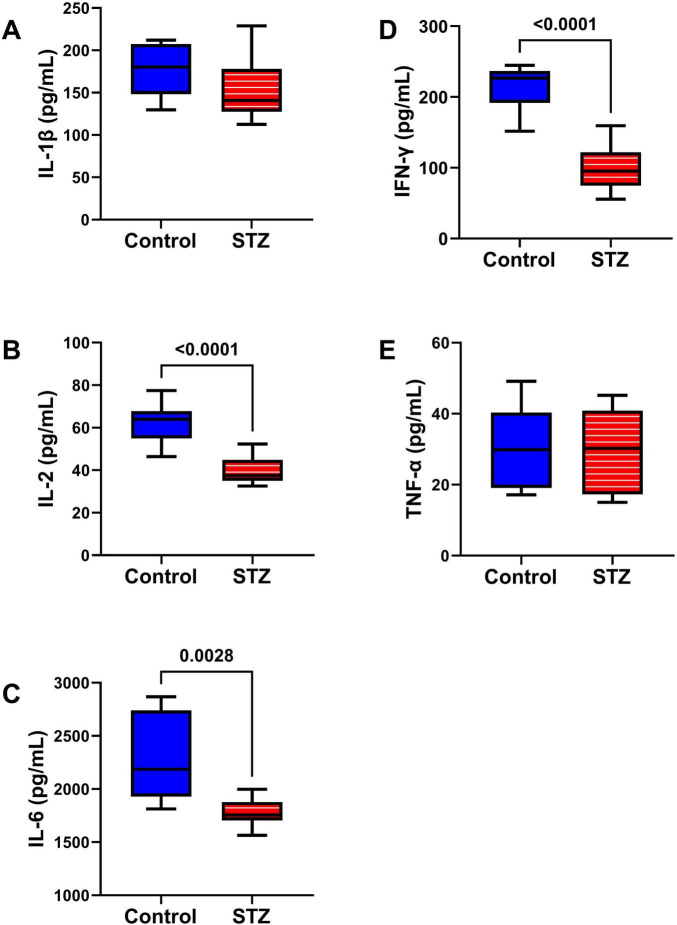
Pro-Inflammatory Cytokines. A multiplex ELISA panel was used to measure the effects of ic-STZ on **(A)** IL-1β, **(B)** IL-2, **(C)** IL-6, **(D)** IFN-γ, and **(E)** TNF-α immunoreactivity in temporal lobe tissue (*n* = 8 controls and 11 ic-STZ rats). Inter-group comparisons were made by repeated measures *t*-tests. Significant (*p* ≤ 0.05) differences are displayed.

Inflammatory Cytokines Studies: A cytokine-focused multiplex ELISA measured TL levels of IL-1β, IL-2, IL-6, IFN-γ, and TNF-α (Figure 7). Although neuroinflammation is implicated in the pathogenesis of AD and other neurodegenerative diseases (Rubio-Perez and Morillas-Ruiz, 2012), this study demonstrated significant ic-STZ-associated reductions in IL-2 (Figure 7B), IL-6 (Figure 7C), and IFN-γ (Figure 7D), but no detectable effects on IL-1β (Figure 7A) or TNF-α (Figure 7E).

**FIGURE 8:**
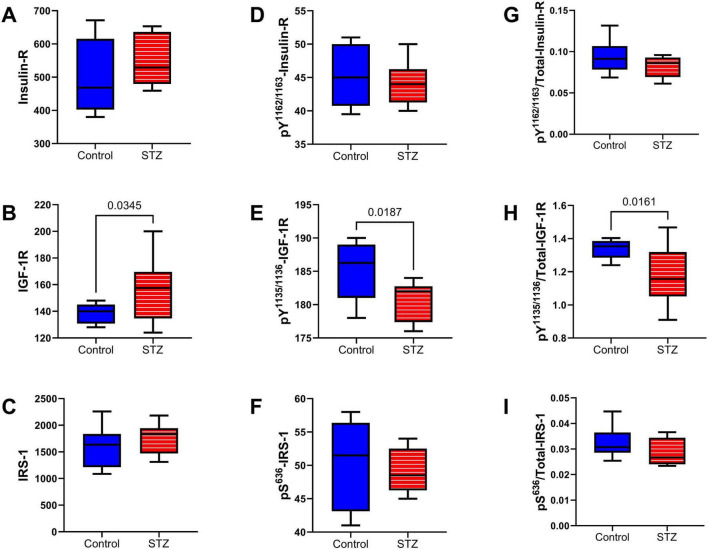
Upstream signaling molecules. The effects of ic-STZ on temporal lobe expression of **(A)** Insulin receptor (Insulin-R), **(B)** IGF-1R, **(C)** IRS-1 **(D)** ^pY 1162/1163^-Insulin-R **(E)** ^pY 1135/1136^-IGF-1R, **(F)** ^pS 636^-IRS-1 were examined using commercial multiplex bead-based ELISA platforms. The **(G)** ^pY 1162/1163^/T-Insulin-R, **(H)** ^pY 1135/1136^/T-IGF-1R, and **(I)** ^pS 636^/T-IRS-1 calculated ratios correspond to relative levels of signaling molecule phosphorylation. Inter-group comparisons (*n* = 8 control and 11 ic-STZ rats) were made by repeated measures *t*-tests. Significant (*p* ≤ 0.05) differences are displayed.

Upstream Insulin/IGF-1 and IRS-1 Signaling Pathway: Insulin receptor (Insulin-R), insulin-like growth factor, type 1 receptor (IGF-1R), and insulin receptor substrate, type 1 (IRS-1) total (Figures 8A–C) and phosphorylated (Figures 8D–F) protein expression were measured by multiplex ELISA. Their calculated levels of relative phosphorylation were also assessed. The studies demonstrated a significant increase in the mean level of IGF-1R (Figure 8B) but significantly reduced levels of ^pY1135/1136^-IGF-1R (Figure 8E) and p/T-^pY1135/1136^-IGF-1R (Figure 8H) in the ic-STZ samples. In contrast, there were no significant effects of ic-STZ on total Insulin-R (Figure 8A), ^pY1162/1163^-Insulin R (Figure 8D), p/T-^pY1162/1163^-Insulin R (Figure 8G), IRS-1 (Figure 8C), ^pS636^-IRS-1 (Figure 8F), or p/T-^pS636^-IRS-1 (Figure 8I).

**FIGURE 9:**
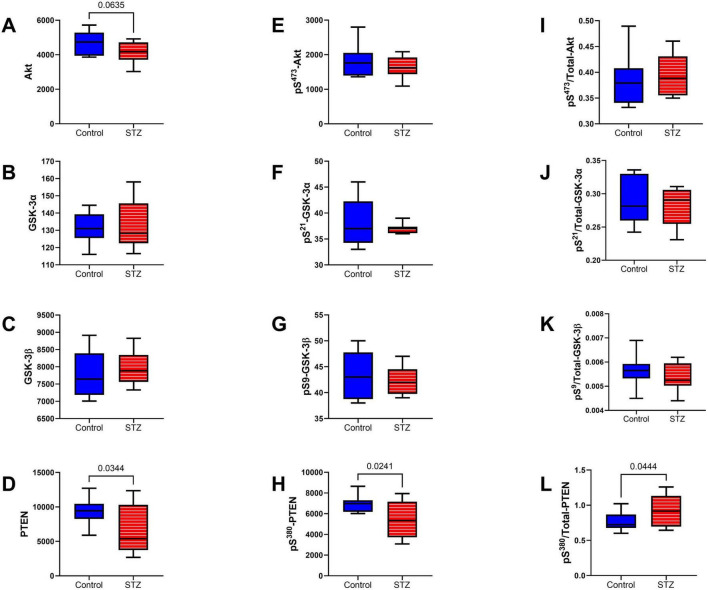
Intermediate signaling molecules. The effects of ic-STZ on temporal lobe expression of **(A)** Akt, **(B)** GSK-3α, **(C)** GSK-3β, **(D)** PTEN, **(E)** ^pS 473^-Akt, **(F)** ^pS 21^-GSK3α, **(G)** ^pS 9^-GSK3β, **(H)** ^pS 380^-PTEN were measured with total and phospho-protein multiplex ELISA platforms. The **(I)** ^pS 473^/T-Akt, **(J)** ^pS 21^/T-GSK3α, **(K)** ^pS 9^/T-GSK3β, and **(L)** ^pS 380^/T-PTEN calculated ratios correspond to relative levels of signaling molecule phosphorylation. Inter-group comparisons (*n* = 8 control and 11 ic-STZ rats) were made by repeated measures *t*-tests. Significant (*p* ≤ 0.05) and statistical trend-wise (0.05 < *p* < 0.10) differences are displayed.

Intermediate Signaling Molecules: The multiplex ELISA panels measured the total (Figures 9A–D) and phosphorylated (Figures 9E–H) levels of Akt, GSK-3α, GSK3β, and PTEN, and those results were used to calculate the relative levels of protein phosphorylation (p/T) (Figures 9I–L). Significant effects of ic-STZ were marked by reduced levels of PTEN (Figure 9D) and ^pS380^-PTEN (Figure 9H), and increased levels of p/T-^pS380^-PTEN (Figure 9L). In addition, total Akt exhibited a statistical trend-wise reduction related to ic-STZ (*p* = 0.06; Figure 9A). There were no significant effects of ic-STZ on TL levels of ^pS473^-Akt (Figure 9E), p/T- ^pS473^-Akt (Figure 9I), or the total, phosphorylated or relative phosphorylated GSK-3α (Figures 9B, F, J) and GSK3β (Figures 9C, G, K).

**FIGURE 10:**
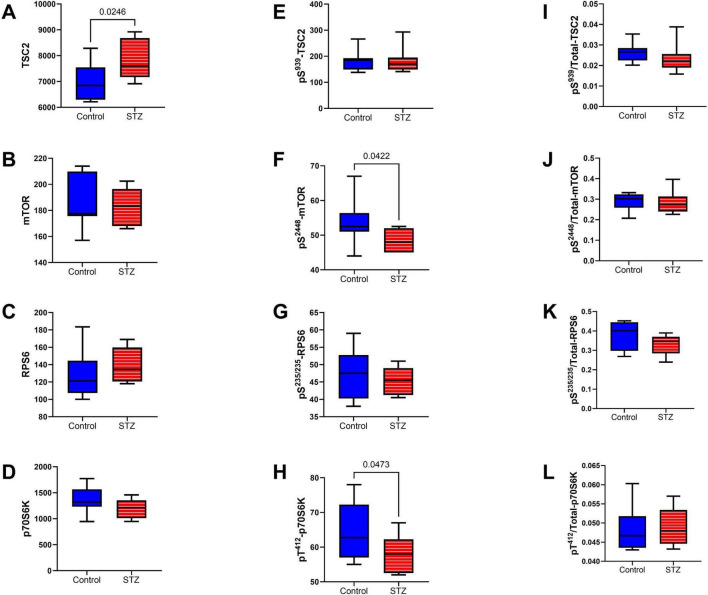
mTOR pathway signaling. Immunoreactivity to **(A)** TSC2, **(B)** mTOR, **(C)** RPS6, **(D)** p70S6K, **(E)** ^pS 939^-TSC2, **(F)** ^pS 2448^-mTOR, **(G)** ^pS 235/236^-RPS6, **(H)** ^pT 412^-p70S6K were measured with total and phospho-protein multiplex ELISAs. Relative levels of phosphorylation represented by **(I)** ^pS 939^-/T-TSC2, **(J)** ^pS 2448^/T-mTOR, **(K)** ^pS 235/236^/T-RPS6, and **(L)** ^pT 412^/T-p70S6K were calculated from the ratios of phosphorylated to total protein immunoreactivity. Inter-group comparisons (*n* = 8 control and 11 ic-STZ rats) were made by repeated measures *t*-tests. Significant (*p* ≤ 0.05) differences are displayed.

**FIGURE 11:**
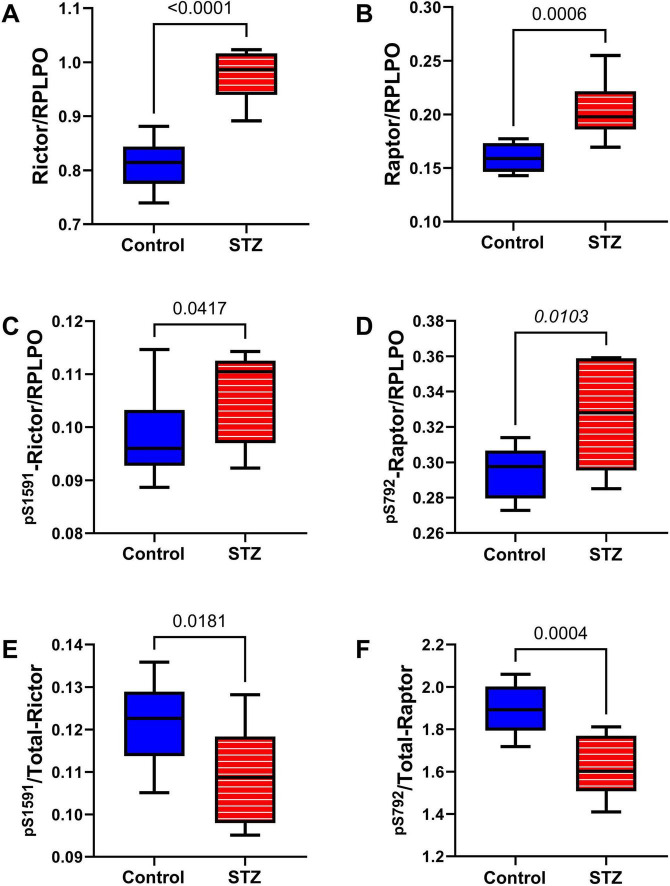
Rictor/Raptor Adaptor Molecules. Duplex ELISAs were used to measure temporal lobe immunoreactivity to **(A)** Rictor, **(B)** Raptor, **(C)** ^pS 1591^-Rictor, and **(D)** ^pS 792^-Raptor with results normalized to RPLPO. **(E)** ^pS 1591^/T-Rictor and **(F)** ^pS 792^/T-Raptor, corresponding to the relative levels of phosphorylation, were calculated from ratios of phosphorylated to total protein. Inter-group comparisons (*n* = 8 control and 11 ic-STZ rats) were made by repeated measures *t*-tests. Significant (*p* ≤ 0.05) differences are displayed.

mTOR pathway: Included among the mTOR pathway molecules evaluated using total and phosphoprotein multiplex ELISAs were TSC, mTOR, RPS6, and p70S6k (Figure 10). Significant ic-STZ effects were shown by the increased TSC2 (Figure 10A), and reduced ^pS2448^-mTOR (Figure 10F), and ^pT412^-p70S6K (Figure 10H). In contrast, no significant effects of ic-STZ were observed with respect to ^pS939^-TSC2 (Figure 10E), p/T ^pS939^-TSC2 (Figure 10I), mTOR (Figure 10B), p/T- ^pS2448^-mTOR (Figure 10J), RPS6 (Figure 10C), ^pS235/236^-RPS6 (Figure 10G), p/T-^pS235/236^-RPS6 (Figure 10K), p70S6K (Figure 10D), or p/T- ^pT412^-p70S6K (Figure 10L). Additional duplex ELISAs were performed to measure the ic-STZ effects on Rictor and Raptor, the main regulators of mTORC complex and mTOR signaling (Rosner et al., 2010). The effects of i.c STZ on total (Figures 11A, B), phosphorylated (Figures 11C, D), and the relative phosphorylated (Figures 11E, F) levels of Rictor and Raptor were virtually identical in that all inter-group differences were statistically significant and marked by increased total and phosphorylated signaling molecule expression but reduced relative levels of phosphorylated Rictor and Raptor in the ic-STZ-treated group.

## 4 Discussion

This study assessed the integrity of mTOR signaling networks in the temporal lobes of an ic-STZ model with AD-type neurodegeneration and builds on prior evidence that AD is integrally linked to dysregulated signaling through the insulin and/or IGF receptors, and downstream through PI3K-Akt with activation of GSK-3β (de la Monte and Wands, 2005; Rivera et al., 2005; Steen et al., 2005; Talbot et al., 2012; Hoyer, 2004). Consequences include impairments in glucose utilization, energy metabolism, neuronal survival and plasticity, and homeostatic mechanisms, combined with increased oxidative stress and mitochondrial dysfunction (de la Monte, 2023; Cunnane et al., 2020; de la Monte, 2014; de la Monte and Wands, 2005). These pathophysiological processes contribute to cognitive decline and brain accumulations of signature hyperphosphorylated Tau-associated paired-helical filament lesions, i.e., neurofibrillary tangles, dystrophic neurites, and neuropil threads, and Aβ-containing senile plaques and fibrils (de la Monte, 2014; Gabbouj et al., 2019; Li et al., 2022). However, the need to pursue additional mechanistic studies is underscored by the lack of effective treatments and persistent incomplete understanding of the full spectrum of AD pathology, including white matter degeneration with myelin, fiber, and oligodendrocyte loss, which contributes to cognitive decline and dementia (Tahami Monfared et al., 2022; de la Monte, 2023; Cunnane et al., 2020). A more detailed understanding of dysregulated brain metabolism in AD could lead to better treatments based on expanded targeting of signal transduction networks, including those needed to support white matter integrity. The approach was to re-evaluate the neurobehavioral and AD-type neurodegenerative effects of ic-STZ sporadic AD model in relation to long-term dysregulation of brain insulin/IGF signaling and its crosstalk with the mTOR pathway. Importantly, mTOR signaling has pivotal roles in oligodendrocyte functions needed to maintain white matter integrity, bioenergetics, and neuroprotection (Figlia et al., 2018; Narayanan et al., 2009; Carson et al., 2015; Grier et al., 2017; Tyler et al., 2009).

STZ is a nitrosamine-related toxin used to generate preclinical models of diabetes mellitus (Goyal et al., 2016), but its application to studies of neurodegeneration helped direct mechanistic analyses of AD to impairments in glucose utilization and energy metabolism as mediators of cognitive impairment (Salkovic-Petrisic et al., 2006; de la Monte et al., 2017). As a glucosamine nitrosourea, STZ, in effect, substitutes for glucose and accumulates intracellularly via the GLUT2 transporter. STZ exerts its cytotoxic effects as an alkylating agent that causes DNA damage, DNA methylation, oxidative stress, and metabolic dysregulation with the accumulation of advanced glycation end-products (Goyal et al., 2016). Although STZ prominently targets pancreatic Beta cells responsible for insulin production, its broad use in the generation of “sporadic” AD-type models of neurodegeneration has broadened the mechanistic understanding of how insulin resistance and insulin deficiency disease states contribute to AD pathogenesis and inspired the development of novel treatment strategies (de la Monte et al., 2017; Moreira-Silva et al., 2019; Lopez Hanotte et al., 2024; Santos et al., 2012; Shingo et al., 2012; Shingo et al., 2015; Sun et al., 2018; Tota et al., 2011). The latter point is especially of interest in regard to the demonstration that incretin agonists can significantly ameliorate STZ-mediated AD-type neurodegeneration and cognitive impairment in experimental models (Paladugu et al., 2021; Patel et al., 2022; Kang et al., 2023; Siddiqui et al., 2023; Zago et al., 2024) vis-à-vis strong evidence for similar pathologies in human AD brains (de La Monte, 2024; de la Monte et al., 2019; Robbins et al., 2020). For this research, we treated young rats with ic-STZ to generate a robust model of AD-type neurodegeneration with significant neurobehavioral deficits. The substantial performance impairments on the Morris water maze and rotarod tests reflect deficits in spatial learning and memory and motor dysfunction as previously reported (Lester-Coll et al., 2006; Moreira-Silva et al., 2019; Tong et al., 2016b). Correspondingly, other studies utilizing the ic-STZ model also demonstrated significant deficits in neurobehavioral function that correlated with neurodegeneration, including Barnes maze, novel object recognition, and plus maze performance, and were unrelated to slow motor performance or weakness (Lester-Coll et al., 2006; Moreira-Silva et al., 2019; Tong et al., 2016b; Lopez Hanotte et al., 2024; Santos et al., 2012; Shingo et al., 2012; Zappa Villar et al., 2018; Riberas-Sanchez et al., 2024). Although impairments in Morris water maze performance are often attributed to damage in the temporal lobes and hippocampi (Gerlai, 2001), other contributing pathologies occur in white matter and the frontal lobes (Homans et al., 2022). Similarly, rotarod performance deficits often mark cerebellar abnormalities (de la Monte et al., 2011; Goodlett et al., 1991), but cerebellar atrophy, neurodegeneration, and dysfunction are recognized components of AD (Chen et al., 2024; Gellersen et al., 2017; Sepulveda-Falla et al., 2014; Fukutani et al., 1997), has been reported previously in the ic-STZ model (Tong et al., 2016a; Shingo et al., 2015), and contributes to cognitive impairment (Brandt et al., 2004), including deficits in spatial learning and memory (D’Hooge and De Deyn, 2001).

The increased terminal body and liver weights in ic-STZ-treated rats were likely manifestations of insulin resistance despite non-elevated blood glucose. Similar effects of ic-STZ have been reported previously (Lester-Coll et al., 2006; Tong et al., 2016b). Although the absence of elevated blood glucose indicates that the ic-STZ rats were not diabetic, previous studies showed that STZ causes liver injury with inflammation and hepatic steatosis, mimicking non-alcoholic fatty liver disease (Kohl et al., 2013; Yazdi et al., 2019). Similarly, other experimental nitrosamine exposures were shown to cause peripheral and hepatic insulin resistance and inflammation, and liver enlargement with hepatic steatosis in connection with neurodegeneration (de la Monte et al., 2009b; Tong et al., 2009; Tong et al., 2015; Zabala et al., 2015). Although hepatic and systemic factors were not evaluated in the present study, it remains conceivable that the nitrosamine-type toxic effects ultimately led to increased body and liver weights in the ic-STZ-treated rats. Of note is that at the times of rotarod and Morris water maze testing, the mean body weight of the ic-STZ group was not elevated. Therefore, excessive weight could not account for the compromised neurobehavioral performance of the ic-STZ-treated rats. However, during the final week of the experiment, after the neurobehavioral testing had been completed, the ic-STZ group gained weight, accounting for their significantly higher terminal mean body and liver weights, and likely marking the onset of systemic and hepatic insulin resistance. An important limitation of this study is that a detailed longitudinal analysis of systemic and CNS physiological and molecular ic-STZ-related pathologies, including time points beyond the planned experiment, was not performed.

In contrast, the brain weight was significantly reduced in the ic-STZ-treated rats. Our studies focused on neuropathological and related molecular and biochemical abnormalities in the temporal lobes because, like AD in humans, ic-STZ causes prominent neurodegeneration with dysregulated metabolism, oxidative stress, (Lester-Coll et al., 2006; Moreira-Silva et al., 2019; Tong et al., 2016a; Tong et al., 2016b; Santos et al., 2012; Shingo et al., 2012; Tota et al., 2011; Zappa Villar et al., 2018; Riberas-Sanchez et al., 2024). Correspondingly, the neuropathological effects of ic-STZ reported herein confirm that the temporal lobe and hippocampi are targets of metabolic dysregulation mediated by insulin/IGF-signaling due to ic-STZ neurotoxicity and neurodegeneration. One potential explanation for why ic-STZ targets the temporal lobes is that insulin and IGF signaling mediators are abundantly expressed in temporal lobe structures, as well as the cerebellar cortex (de la Monte, 2014; de la Monte and Wands, 2005), which we also showed exhibited neurodegeneration in our model. The associated atrophy of the temporal lobes, including cortex, white matter, and hippocampi, corresponded with the Morris water maze performance deficits, consistent with prior observations (Tong et al., 2016b; Ishrat et al., 2009). The ongoing neurodegenerative changes (neuronal condensation, darkening, shrinkage, and apoptosis) illustrated in hippocampal and cortical neurons most likely reflect ongoing increased vulnerability to oxidative stress, including the effects of terminal anesthesia. Striking atrophy and degeneration of the temporal lobe and hippocampal formation have been well-documented in previous reports (Lopez Hanotte et al., 2024; Santos et al., 2012; Shingo et al., 2012; Zappa Villar et al., 2018; Riberas-Sanchez et al., 2024). In addition, there is evidence that temporal lobe/hippocampal neuronal loss may be mediated by impaired neurogenesis (Sun et al., 2018). The ic-STZ-associated white matter atrophy and myelin pallor illustrated with the Luxol fast blue stain reflects loss of myelin and axons, like the findings previously observed in humans with AD (de la Monte, 2023; Bloom, 2014). The findings of increased temporal lobe pTau, pTau/Tau, Aβ, and AChE immunoreactivities support the concept that the ic-STZ model is reminiscent of sporadic AD. The impairments in insulin/IGF signaling with attendant dysregulation of energy metabolism led to increased GSK-3β activity and oxidative stress and thereby contributed to AD-type pathologies, including cholinergic deficits marked by the increased AChE expression (Salkovic-Petrisic et al., 2006; García-Ayllón et al., 2011).

The elevated level of the lipid peroxidation marker, HNE, highlighted a mechanism of ongoing tissue injury, weeks after the ic-STZ treatment. Elevated HNE can mediate damage to mitochondrial DNA, decrease energy production (Bekyarova et al., 2019), exacerbate Aβ aggregation and Tau hyperphosphorylation (Butterfield et al., 2001; Liou et al., 2019). Furthermore, HNE is known to be elevated in AD and other neurodegenerative diseases (Li et al., 2022). The reduced ubiquitin in ic-STZ temporal lobe samples may seem paradoxical given the known AD-associated accumulations linked to misfolded protein aggregates (Amzallag and Hornstein, 2022; Behl et al., 2022; Tecalco-Cruz et al., 2022; Tokunaga and Ikeda, 2022), However, the finding could be explained by proteasome inactivation vis-à-vis extreme oxidative stress ultimately compromising the clearance of damaged proteins (Oddo, 2008; Löw, 2011) while reducing newly formed ubiquitin conjugates needed for the degradation of abnormal proteins (Shang and Taylor, 2011).

The ic-STZ-associated reductions in temporal lobe pro-inflammatory cytokine markers on the surface are contrary to expectations based on the widely accepted role of neuro-inflammation in AD (Rubio-Perez and Morillas-Ruiz, 2012; Park et al., 2020; Kinney et al., 2018). However, emerging data suggest that pro-inflammatory states in AD may not represent a constant or continuous process within and across studies, and in relation to disease progression (Park et al., 2020). For example, patients with moderate AD were found to have reduced plasma levels of multiple pro-inflammatory cytokines (Koca et al., 2022). Consideration has been given to the potential roles of extrinsic, non- central nervous system (CNS) factors, e.g., the brain-gut axis, as a mediator of peripheral immune cellular activation and cytokine secretion in AD and other forms of neurodegeneration (Park et al., 2020). Finally, genetic polymorphisms may contribute to the nature of pro-inflammatory responses in AD (Su et al., 2016). With regard to the ic-STZ model, in the early stages following STZ treatment (de la Monte et al., 2017; Santos et al., 2012), increased inflammation is a consistent occurrence with elevations in tissue or CSF and plasma pro-inflammatory cytokines (Lee et al., 2016; Mensah-Brown et al., 2005), similar to the findings in the early stages of AD (de la Monte et al., 2023b; Lee et al., 2013). However, later reductions in cytokine activation have been reported and attributed to depletion of T regulatory cells (Lee et al., 2016).

Although the inhibitory effects of ic-STZ could have reflected inhibition of pro-inflammatory responses, they may instead have corresponded to non-inflammatory functions of these molecules in the CNS. For example, IL-2 has neuroprotective actions that help maintain cholinergic septo-hippocampal neurons in the CNS, whereas in the periphery, the same cytokine enhances T cell proliferation (Meola et al., 2013). Of interest in this regard is that IL-2 expression in AD hippocampal biopsies was found to be reduced (Riberas-Sanchez et al., 2024), suggesting a role in mediating cholinergic deficits linked to cognitive impairment and neurodegeneration in the early stages of AD (García-Ayllón et al., 2011; Ferreira-Vieira et al., 2016). In the CNS, IFN-γ also has either pro-inflammatory or neuroprotective actions that shift with expression levels and disease stage (Ottum et al., 2015). Since IFN-γ-mediated neuroprotection supports neurogenesis effects that augment spatial learning and memory (Baron et al., 2008), in that capacity, its significantly reduced expression in the temporal lobes of ic-STZ-treated rats may have contributed to impaired performance on the Morris water maze test. Finally, pro-inflammatory IL-6 is up-regulated in AD brains and plasma, negatively correlating with cognitive and peripheral metabolic impairment (Lyra et al., 2021), yet evidence suggests its actions include anti-inflammatory and neuroprotective functions (Garcia-Juarez and Camacho-Morales, 2022). The factors controlling the pro- versus anti-inflammatory status of these traditional cytokines in the CNS are still under consideration. However, their diametrically opposing roles as drivers of tissue injury versus neuroprotection challenge traditional therapeutic interventional concepts.

The early effects of ic-STZ include inhibition of insulin and IGF signaling networks from proximal points at receptor tyrosine kinase levels downstream through IRS and then PI3K-Akt (Lester-Coll et al., 2006; Salkovic-Petrisic et al., 2006; Moreira-Silva et al., 2019; Kadowaki et al., 1984). Previous studies showed that ic-STZ-inhibits frontal lobe expression of insulin/IGF-1-Akt-mTOR pathway signaling molecules (Lester-Coll et al., 2006), whereas in other studies, the main effects of ic-STZ in the temporal lobe included predominantly inhibitory effects on IGF-1 receptor rather than insulins (de la Monte et al., 2017), as observed herein. Although the explanation is not fully evident, the findings may reflect inherent regional differences in receptor vulnerability. Alternatively, given the relatively greater importance of IGF-1 compared with insulin signaling in oligodendrocytes (Wrigley et al., 2017), the more prominent long-term inhibitory effects of ic-STZ on IGF-1 receptor signaling may correspond with the reductions in white matter myelin and oligodendrocyte density in the corpus callosum (See Figure 4). Importantly, the insulin and IGF-1 receptors share a high degree of homology and both have pivotal roles in regulating cellular metabolism and growth (Alves et al., 2016). In aggregate, the statistical trend-wise reduction in ^pY 1162/1163^/Total-Insulin-R, and significant reductions in ^pY 1135/1136^-IGF-1R and ^pY 1135/1136^/Total IGF-1R indicate that ic-STZ had long-term inhibitory effects on the most upstream components of the insulin/IGF-1 metabolic pathways in the temporal lobe (Kadowaki et al., 1984; Zhang et al., 1991).

Reduced insulin/IGF-1 receptor signaling can result in decreased tyrosine phosphorylation of IRS-1 with attendant inhibition of PI3K/Akt (Costa et al., 2012). A limitation of the study was that instead of tyrosine phosphorylation, the multiplex ELISA’s phospho-protein panel assessed ^pS 636^-IRS-1, which has inhibitory effects on insulin signaling (Talbot et al., 2012; Aguirre et al., 2002; White, 2002) and is regulated by pro-oxidants such as TNF-α and not insulin/IGF-1 receptor tyrosine kinases (Peraldi et al., 1996). The findings did not reveal any alterations in IRS-1 or ^pS 636^-IRS-1 in the temporal lobes of ic-STZ-treated rats. Correspondingly, apart from the trend-wise inhibition of Akt, there were no significant alterations in the Akt/GSK-3α/GSK-3β total or phospho-protein expression related to ic-STZ treatment. While the significantly reduced level of PTEN phosphorylation would have promoted Akt inhibition and GSK-3 activation (de la Monte, 2023), the reduced level of PTEN protein and its relatively increased phosphorylation would have favored the dis-inhibition of Akt. In essence, the aggregate long-term net effect of the ic-STZ on Akt, GSK-3, and PTEN appears to have been neutral. The compensatory cellular mechanisms utilized to apparently normalize this intermediate level of signaling molecule expression are not immediately evident, but the consequences are likely related to the maintenance of function adequate for survival despite cognitive-motor impairments measured by Morris water maze and rotarod testing.

The main long-term effects of ic-STZ on metabolic signaling significantly impacted mTOR. The inhibitory effects of ic-STZ on mTOR were evidenced by the significantly elevated level of TSC2, which inhibits mTOR/mTORC signaling (de la Monte, 2023). Correspondingly, serine phosphorylated mTOR, which marks its activated state (de la Monte, 2023), was significantly reduced in the ic-STZ-treated temporal lobes. The ic-STZ treatments significantly increased temporal lobe levels of Rictor (rapamycin-insensitive companion of mTOR) and Raptor (Rapamycin-sensitive companion of mTOR), which are critical components of the mTORC1/2 complexes and serve to stimulate downstream signaling through p70S6K and RPS6 (Rosner et al., 2010; Biever et al., 2015; Liu et al., 2015). However, those potentially compensatory responses were abrogated by corresponding increases in Serine phosphorylated levels of Rictor and Raptor, which would have inhibited mTORC (Querfurth and Lee, 2021). Altogether, the net effect of ic-STZ was to significantly inhibit mTOR/mTORC signaling through p70S6K, and possibly through RPS6 as well. Similarly, in other experimental models of AD, hippocampal mTOR and p70S6K were found inhibited (Han et al., 2016; Liu et al., 2015).

Intact signaling through mTOR/mTORC/p70S6K has critical roles in maintaining white matter integrity, oligodendrocyte function, cell survival, mitochondrial function, RNA translation, and neuronal plasticity (Rosner et al., 2010; Biever et al., 2015; de la Monte et al., 2023a; Harada et al., 2001). Inhibition of signaling through mTOR/mTORC leads to oligodendrocyte dysfunction and deficits in myelination/myelin homeostasis (Carson et al., 2015; Grier et al., 2017; Tyler et al., 2009). In addition, disruption of this pathway likely contributes to the increased tau phosphorylation, pTau aggregation, pro-apoptosis mechanism activation, impaired cell survival and neuronal plasticity, and Aβ accumulation (Han et al., 2016; Liu et al., 2015; Querfurth and Lee, 2021). Correspondingly, the impairments in neurobehavioral functions, neuropathological findings in neurons and white matter, and the associated molecular and biochemical abnormalities detected in the ic-STZ-temporal lobes and hippocampi could be attributed to the inhibition of IGF-1 receptor signaling through mTOR/mTORC pathways. At the same time, future studies should dissect the nature and mechanisms by which, in the face of ongoing neurodegenerative processes, endogenous adaptive neuroprotective mechanisms compensate to broadly sustain brain structure and function, albeit compromised. The lessons learned could inform about novel strategies for reducing or stabilizing the effects of AD and other types of neurodegenerative diseases. As noted earlier, an important limitation of these analyses is that the data were obtained at a single terminal experimental endpoint and only with temporal lobe tissue. Although the intention of the experimental design was to focus on one brain region and time point close to the analysis of naïve performance on neurobehavioral tasks, the relatively narrow scope of investigation restricted the opportunity to understand dynamic shifts in metabolic and neuroinflammatory molecule expression that evolve over time and across different brain regions. Future studies will address these considerations utilizing longitudinal and multiple brain region analytical approaches.

## Data Availability

The raw data supporting the conclusions of this article will be made available by the authors, without undue reservation.
