# Supplementary figures and images for: Dysregulated mTOR networks in experimental sporadic Alzheimer’s disease
Source: Front Cell Neurosci. 2024 Sep 25;18:1432359. doi: 10.3389/fncel.2024.1432359 (PMC11461251; doi:10.3389/fncel.2024.1432359)

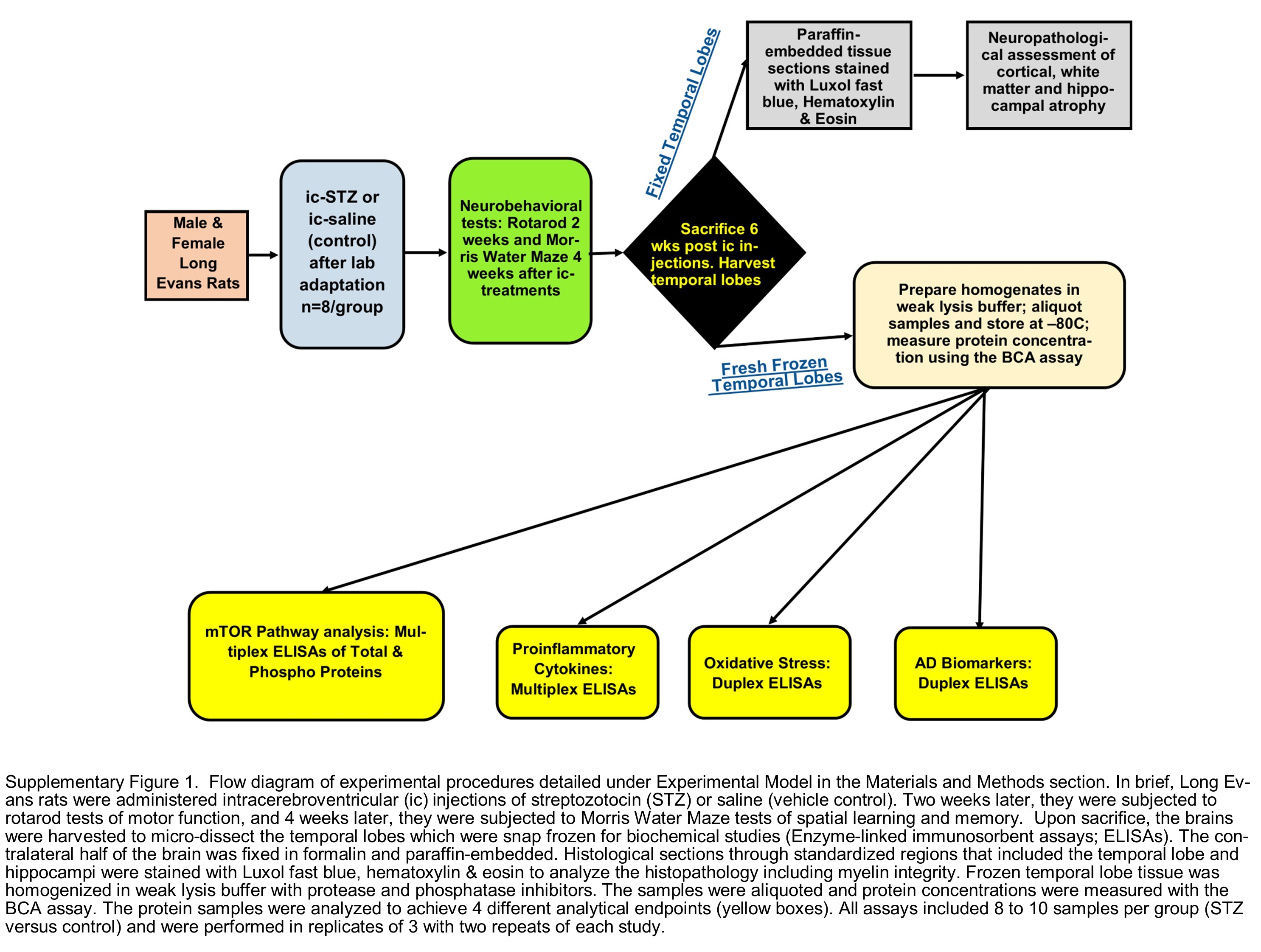

Supplement: Supplementary file 1 [file Image_1.JPEG]
